# Anesthesia, or the Employment of Chloroform and Ether in Surgery, Midwifery, Etc. Etc.

**Published:** 1849-08

**Authors:** 


### REVIEWS.

Art. VII.—*Anesthesia, or the Employment of Chloroform and Ether in Surgery, Midwifery, etc. etc.* By J. T. Simpson, M. D., F. R. S., E. Prof. Midwifery in the University of Edinburg, Physician Accoucheur to the Queen in Scotland, &c. &c. 1 vol. 8vo. pp. 248. Philadelphia, Lindsay and Blakiston, 1849.

Medicine is eminently a progressive science. From the time when man's ingenuity first lent a helping hand to his benevolence, in order to devise means for the relief of suffering humanity, down to the present day, there has been making a constant, untiring series of efforts to extend the boundaries of this noble science, and widen its sphere of usefulness. Success triumphant, though not complete, has crowned these efforts. Medicine, in its primal stage, wrapped in the swaddling clothes of rude simplicity and dubious utility, is now of lofty stature and stands erect—a science built upon truths and principles as immutable as the eternal hills—and an art conferring in the daily exercise of it, untold benefits and countless blessings to thousands. Its progress has been slow, but sure. Thousands of years have rolled away since the sprouting of its germ—and none can say how many more thousands must elapse before the tree has attained its complete growth. But its fruit, if not entirely matured, is already sweet to the taste and invigorating to the body—and year by year, month by month, and day by day, it is becoming sweeter and more invigorating.

Certainly, practical medicine has greatly improved since the days of Hippocrates, and undoubtedly the modern surgeon is far superior to the self-important individual who formerly held the united office of surgeon, cupper, leecher, bleeder, dentist and barber. Yet all this superiority failed in doing more than mitigating the sufferings and the pain of those who required the surgeon's aid. For a long time it had been the darling dream of those who, in the use of instruments, daily witnessed so much suffering, that perhaps a means of avoiding it would one day be found. The desire at length has been gratified; the vision has come to pass; the dream has been realized.

In regard to the history of the discovery of anesthetic agents—to whom belongs the honor of having first found out their efficacy and brought them into notice—it is not our purpose now to speak. Their *practical utility* is a matter of far more importance; and Dr. Simpson's work forms a suitable foundation whereon to build the few remarks we have to offer on this point.

When a new remedy is proposed to the profession as worthy their adoption, two questions instantly arise—questions which must be answered in the affirmative, before its claims can be universally or even generally recognized. These are, 1st. Is it adequate in any degree to meet the ends proposed? And 2d. Is it safe? Let us apply these questions to the use of anesthetics.

1st. Are they adequate in any degree to meet the ends proposed? What is expected to be gained by their use? Their advocates say, and challenge investigation, that these agents are capable of wholly destroying the pain attendant on surgical operations, in a large majority of cases, and greatly mitigating it in all. This, if true, is not the "trivial matter" that Majendie and other opponents of etherization would have us believe; and that it is true, few will now have the hardihood to deny. The voice of those members of the profession who have given it a fair trial in a proper way, is so unanimous in its testimony as to this point; and is swollen by such a large and united concourse of those who in their own persons have made use of it, gratefully acknowledging their exemption from suffering—that it would almost be insulting the intelligence of our readers to offer new and repeated proofs. More than two years ago, Dr. Forbes, in a very able article in the British and Foreign Review, wrote as follows—and every operator who uses these agents confirms his account—

For the purpose of obtaining information on all the points of this most interesting subject, we personally questioned all the patients in the London hospitals, who, at the period of our visits, still remained in the wards after the ether operations. They were in all *fifty-four*, and the great majority had been the subjects of capital operations. They were unanimous in their expressions of delight and gratitude at having been relieved from their diseases without suffering. In listening to their reports, it was not always easy to remain unmoved under the influence of the conceptions thereby communicated, of the astonishing contrast between the actual physical condition of the mangled body in its apparent tortures on the operating table of a crowded theatre, and the really happy mental state of the patient at the time. The old story of the magician in the Arabian Tales seemed more than realized before us, the ether being like the tub of water, one moment's dip of the head into which produced a lifelong vision in the dreamer's mind.

But, furthermore: it is asserted that not only does the use of anesthetics abolish the pain attendant on surgical operations, which, we repeat, is no "trivial matter," but that it conduces largely to the restoration of the patient, and gives him a better chance of ultimate recovery. The proof of this is two-fold. First, the authority of a multitude of intelligent, experienced and candid observers concurring in the assertion, that after severe operations the constitutional disturbance is less and convalescence more rapid in patients who have been etherized, than in those who have not: and second, statictics of a sufficient number of cases to render this view of the case extremely *probable*, though, of course, not absolutely certain.

Chapter V of the work now under notice, is headed "Value and Necessity of the Numerical Method of Investigation as applied to surgery"—and chapter VI contains some interesting statistics in regard to etherization. We wish we could transfer both of them in full to our pages—we are sure they would amply repay perusal; but we must content ourselves with the concluding pages of the latter:

No. VI.—*Table of the Mortality of* 618 *Amputations of the Thigh, Leg, and Arm, without Etherization, performed during the last few years in* 30 *British Hospitals.*

| Seat of Amputation. | PRIMARY. | | | SECONDARY. | | |
|---|---|---|---|---|---|---|
| | No. of Cases. | No. of Deaths. | Per-centage of Deaths. | No. of Cases. | No. of Deaths. | Per-centage of Deaths. |
| Thigh, - | 73 | 45 | 63 | 211 | 62 | 29 |
| Leg, - - | 80 | 26 | 32 | 135 | 23 | 17 |
| Arm, - - | 77 | 17 | 22 | 42 | 10 | 24 |
| TOTAL, - | 230 | 88 | 38 | 388 | 95 | 24 |

GENERAL MORTALITY OF AMPUTATIONS OF THE THIGH, LEG, AND ARM, UPON PATIENTS IN AN ETHERIZED STATE.

In the preceding lengthened Table, No. I, (p. 72,) I have given from forty-nine different hospitals the detailed reports of 302 amputations of the thigh, leg, and arm.

When these 302 amputations are reduced into a tabular form, similar to those which I have used for stating the data of similar amputations without ether, they present the following results:

No. VII.—*Table of the Mortality of* 302 *Amputations of the Thigh, Leg, and Arm, under Etherization.*

| Seat of Amputation. | PRIMARY. | | | SECONDARY. | | |
|---|---|---|---|---|---|---|
| | No. of Cases. | No. of Deaths. | Per-centage of Deaths. | No. of Cases. | No. of Deaths. | Per-centage of Deaths. |
| Thigh, - | 24 | 12 | 50 | 121 | 25 | 20 |
| Leg, - - | 32 | 9 | 28 | 81 | 13 | 16 |
| Arm, - - | 17 | 4 | 23 | 27 | 8 | 29 |
| TOTAL, - | 73 | 25 | 34 | 229 | 46 | 20 |

I shall now proceed to contrast these results with the results of the same operations in the same class of hospitals, and when performed upon patients not in an etherized state.

Before doing so, however, let me observe in passing, that the data I have adduced in Tables No. I. and V., (pp. 72, 76) have been objected to on the ground that they are collected from too many different hospitals, and too many different sources. But, on the contrary, I believe all our highest statistical authorities will hold that this very circumstance renders them more, instead of less trustworthy. Professor Chomel of Paris, after pointing out the first requisite for a successful statistical comparison of therapeutic or other results—viz., a sufficient similarity between the number of collated cases—adds, as the second condition, " that the data be numerous, collected at different times, in different places, and, if possible, by several observers. It is easily seen (he adds) that the results of a number of facts too limited, collected in a short space of time, in a single place, and by a single observer, however exact as regards that individual series of data, may yet be very different from, or even the reverse of conclusions drawn from a larger series, and one collected under various circumstances."

COMPARISON OF THE MORTALITY FOLLOWING THE LARGER AMPUTATIONS OF THE LIMBS, 1. WITHOUT, AND 2. WITH ETHERIZATION.

The major amputations of the limbs, including those of the thigh, leg, and arm, are generally fatal in hospital practice in the proportion of about one in every two or three operated upon. In the Parisian hospitals, the fatality, according to Malgaigne, amounts to upwards of one in two. In Glasgow, it is two and a half. In British hospitals, I found that under these amputations one in three and a half died. The same operations, performed in the same hospitals, and upon the same class of patients, in an anesthetic state, present a mortality of 23 in 100, or 1 in 4, only. The following table shows the amount of the individual cases, and the per-centage of deaths in different collections, with the corresponding proportion of deaths in those operated on in an etherized state.

No. VIII.—*Table of the Mortality of Amputations of the Thigh, Leg, and Arm.*

| Reporter. | No. of Cases. | No. of Deaths. | Per-centage of Deaths. |
|---|---|---|---|
| Parisian Hospitals—Malgaigne, - - - - | 484 | 273 | 57 in 100 |
| Glasgow Hospital—Lawrie, - - - - - | 242 | 97 | 40 in 100 |
| General Collection—Phillips, - - - - - - | 1369 | 487 | 35 in 100 |
| British Hospitals—Simpson, - - - - - | 618 | 183 | 29 in 100 |
| *Upon Patients in an Etherized State,* - - - | 302 | 71 | 23 in 100 |

The evidence which the preceding table affords in favor of the greater safety of amputation with ether than without it, is sufficiently strong and striking. While 23 in 100 died under the amputations named, when the operations were performed upon patients in an anesthetic state, 29 in every 100 died under the same amputations in the same hospitals when the patients were not etherized ;—in the Glasgow hospital as many as 40 in 100 died —and in Paris, as many as 57 per cent. In other words, out of every 100 persons submitted to amputations of the thigh, leg, or arm, the lives of 6 were, by the employment of etherization, saved above the average number of the same operations in British hospitals ;—17 lives in each 100 were saved, if we take the Glasgow returns as a standard of comparison ; the average mortality was, under ether, less by 34 in every 100 cases than that which was found by Malgaigne to accompany the same operations in the Parisian hospitals.

But probably, to most minds, this comparison would be rendered more clear and simple, if we took not a class of operations, but a single operation as a standard and medium of comparison. For this purpose, let us select amputation of the thigh as the *individual* operation regarding which we possess the largest series of observations.

COMPARISON OF THE MORTALITY FOLLOWING AMPUTATIONS OF THE THIGH, 1. WITHOUT, AND 2. WITH ETHERIZATION.

There are few or none of the operations deemed justifiable in surgery, that are more fearfully fatal in their results than amputation of the thigh. "The stern evidence (says Mr. Syme) of hospital statistics shows, that the average frequency of death is not less than from 60 to 70 per cent.;" or above one in every two operated on die. Out of 987 cases of amputation of the thigh collated by Mr. Phillips, 435 proved fatal ; or 44 in every 100 were lost. "On referring (observes Mr. Curling) to a table of amputations in the hospitals of London, performed from 1837 to 1843, collected with care by a private society to which I have the honor of belonging (the Medical Society of Observations), I find 134 cases of amputation of the thigh and leg, of which 55 were fatal, giving a mortality of 41 per cent." Out of 201 amputations of the thigh performed in the Parisian hospitals, and reported by Malgaigne, 126 ended fatally. In the Edinburgh Infirmary 21 died out of 43. Dr. Lawrie found the mortality attendant upon this operation in the Glasgow hospital to amount to 46 deaths in 127 cases. In the collection of cases from 30 different British hospitals which I

have published in Table No. V. (p. 76), 284 cases of amputation of the thigh are reported; 107 out of these 284 operations proved fatal. On the contrary, I have collated 145 cases in which the same operation has been performed during the past year in British hospitals upon patients in an etherized state. Out of these 145 cases of amputation of the thigh, only 37 proved fatal. Or, in other words, the fatality was not greater than one in every four operated on when the patients were previously etherized. It was as high as one in every two or three operated on when the patients were not previously etherized. The following Table presents these results in a more clear form :—

No. IX.—*Table of the Mortality of Amputation of the Thigh.*

| Reporter. | No. of Cases. | No. of Deaths. | Per-centage of Deaths. |
|---|---|---|---|
| Parisian Hospitals—Malgaigne, - - - - | 201 | 126 | 62 in 100 |
| Edinburgh Hospital—Peacock, - - - - | 43 | 21 | 49 in 100 |
| General Collection—Phillips, - - - - - | 987 | 435 | 44 in 100 |
| Glasgow Hospital—Lawrie, - - - - - | 127 | 46 | 36 in 100 |
| British Hospitals—Simpson, - - - - - | 284 | 107 | 38 in 100 |
| *Upon Patients in an Etherized State,* - - - | 145 | 37 | 25 in 100 |

The preceding figures speak in a language much more emphatic than any mere words that I could employ in favor of anesthesia, not only as a means of preserving surgical patients from pain, but as a means also of preserving them from death. Between even the lowest mortality in the table without ether, 32 in 100, and the rate of mortality with it, 65 in 100, there is the difference of 11 per cent. That is to say, according to this standard, out of every 100 patients submitted to amputation of the thigh without anesthesia, 11 more would die from the operation than if the same 100 patients were submitted to the same operation in a state of anesthesia. And if the condition of anesthesia effects thus a saving of 11 lives in every 100 amputations of the thigh;—then out of every 1000 such operations the lives of 110 patients would be preserved by the use of anti-pathic means.

If we compare these results with the standard of Mr.

Phillips, the contrast is still more startling. Out of 987 amputations of the thigh collected by him, 435 proved fatal; or 44 in the 100. Out of 145 amputations of the thigh under anesthesia, 37 proved fatal, or 25 in 100.—According to this comparison, the amount of persons saved from death in amputation of the thigh by the patients being rendered anesthetic during the operation, amounts to 19 lives in every 100 operations performed.

Surely the opponents of etherization should cease their opposition until they can bring *facts* and *figures* to controvert these—instead of the unfounded assertions, hasty deductions and prejudiced opinions which have marked the course of a portion of those who will not adopt this new practice. Others of them, and these doubtless constitute by far the greater portion, both in point of numbers, respectability, and professional eminence, object to the use of anesthetics, because already, in the short time in which they have been in use, quite a number of cases have occurred in which death followed their exhibition; and this brings us at once to the second question raised above, viz: Is the use of these articles safe and prudent?

That ether and chloroform are both most powerful agents, none will deny. That they should be used with caution and only by the physician, their stoutest advocates will freely admit. That they are *dangerous*, in any proper sense of that word, or that the evil accruing from their use in any degree balances the good, they and we do not, and until more and better proof is given than we have yet seen, cannot believe.

Death has, undoubtedly, in more than one instance, followed the employment of these agents—and followed them so instantaneously that there can be no reasonable doubt as to whether or not they were really the producing cause. This result may have been due to one of two things:

1. The impurity of the agent employed.

2. To a peculiar condition, inherent in the constitution and not to be foreseen by any ordinary sagacity, which of necessity brings about this result.

This latter, we think, is the most probable, or rather the most frequent cause of these ill effects. At any rate, for the sake of argument we will admit it to be so. Now, if merely because the systems of a few—a very few—will not bear the exhibition of any given medicine, it must be discarded from the list of remedial agents, many of the most important reliable weapons with which the physician has to combat disease must be thrown aside as worse than useless. Opium, arsenic, digitalis, hydrocyanic acid, calomel, the lancet, &c., &c., must all be dispensed with, because all, in some cases, have done damage. One of these agents—calomel—has repeatedly, over and over again—and that in spite of all reasonable prudence on the part of the administrator—produced the most horrible deformities, the most acute and poignant suffering, and finally death itself: and yet is there an intelligent physician in this country who would dispense with its use on that account? Most assuredly not. And why? The answer is obvious—because the good which it is capable of doing, and does do, far overbalances the evil which it has done, and, used prudently, can do. And so of the others—and why *not* so of ether and chloroform. Apply the same reasoning to them: nay, go farther, and suppose that they alone were capable of occasionally bringing about an unfortunate issue—that the lancet and digitalis and calomel could not with justice be charged with any such result—yet would we not still be justified in the use of them? Let us return for a moment to our statistics. It is admitted on all hands that these agents do produce the effects ascribed to them—viz: the abolition of all sense of pain—nay, frequently lap the patient in perfect elysium, while otherwise he would be suffering the torments of the damned. Is that nothing? We have, too, the united voice of all who have used them, bearing witness to the fact that the recoveries are more rapid than they otherwise would be. Is that nothing? And, to crown all, we have statistical tables based upon the results of a large number of operations—performed, most of them, in hospitals, and hence as nearly as possible under the same circumstances, on the same class of people, by the same operators—showing conclusively that these articles, so far from being *destructive*, are in a high degree *conservative*. Turn back, reader, and ponder over the two concluding paragraphs of the extract we have given from Dr. Simpson's book.

"Between even the lowest mortality," he shows, "in the Table [No. IX.] without ether—36 in 100—and the rate of mortality with it—25 in 100—there is a difference of *eleven per cent*." That is, *etherization saves* 11 *people out of every* 100 *who undergo a capital operation*—110 *out of every* 1000. Does it kill 110 out of every 1000 *who take it?* No. Does it kill 10 in that number? It is not so pretended. Does it kill *one?* Most indubitably *not*.

They who object to the use of it, because it is *unsafe*, have here some food for further reflection.

A medical journal is hardly the most suitable place to discuss the general question of utilitarianism; and if it were, the writer by no means considers himself able to conduct such a discussion—but it does seem to us perfectly plain, that "the greatest good of the greatest number," is an axiom as fairly applicable to *medical* science as any other science, and medical practitioners are as much bound to act upon it as any other class of individuals.

While we have no shadow of right to resort to etherization in any case where serious results may be fairly apprehended, and while it would be nothing more nor less than downright murder to use it where from any circumstances we judged it would be likely to produce the baneful effects ascribed to it; yet surely considerations of high magnitude call for it in a multitude of cases. For, it has been shown that the administration of anesthetics *certainly saves* many more lives than it has been even charged with destroying. And, for our part, it seems to us more reasonable to say to one who refuses, in the performance of capital operations, to resort to these agents, "*You, sir, are responsible for eleven of every hundred deaths that occur in your practice under these circumstances,*" than to lay an accidental occasional misfortune—rare in its occurrence, and by no means always clearly traceable to the employment of these articles—to the door of him who uses them when this use is encouraged by humanity, supported and sustained by the most cheering results, and imperiously demanded by a regard for human life.

The application of these same agents to another very extensive and important branch of practical medicine—obstetrics—is of even later date than that of which we have just been speaking, and has met with still greater opposition.

The same objections meet us here—*inefficiency* and *danger*—that have been so often and so futilely urged against the use of these articles in surgery; and experience already has shown them to be no sounder.

A little more than two years ago, Dr. Simpson, Professor of Midwifery in the University of Edinburg, determined to use, on the first suitable occasion, the vapor of sulphuric ether, in order to try if the same favorable results might not be obtained for the relief of this form of suffering.

The first case in which it was tried, was one in which there was much deformity of the pelvis. Now as the capability of ether to abolish sensibility had been fully proved, and its alleged danger to life disproved, the only question of any practical importance was, would it arrest the contractions of the uterus as well as the sufferings that arise from them? As version would here be required at any rate, it mattered little, so far as this case was concerned, whether it did or not; and therefore it was deemed a most suitable one for the experiment—which was accordingly made, with the most perfect and encouraging success. But little time was allowed to elapse between the moment of rendering the patient unconscious and the completion of the delivery; but during that time the uterine contractions were strong and energetic.

On questioning the patient after her delivery, she declared that she was quite unconscious of pain during the whole period of the turning and extracting of the infant, or indeed from the first minute or two after she commenced to breathe the ether. The inhalation was discontinued towards the latter part of the operation, and her first recollections on awaking were "hearing," but not "feeling," the head of the infant "jerk" from her (to use her own expressions), and subsequently she became more roused by the noise caused by the preparation of a bath for the child. She quickly regained full consciousness, and talked with gratitude and wonderment of her delivery, and her insensibility to the pains of it. Next day I found her well in all respects. I looked in upon her on the 24th (the fifth day after delivery), and was astonished to find her up and dressed, and she informed me that on the previous day she had walked out of her room to visit her mother. Mr. Figg informs me that her further convalescence has been uninterruptedly good and rapid.

Since this case was published, a day or two after, ether and chloroform (the *anesthetic* powers of which were discovered by Dr. Simpson, in his search after some more pleasant and agreeable agent than ether) have been used in an immense number of cases; and *uniformly*, so far as we have been able to learn, with the happiest results. Can as much be said for any other medicine?

The distinguished professor has used it with "*unvarying success:*" and be it remembered, he administers it in

*all* cases of labor, whether natural or preternatural. Drs. Duncan and Norris, house-surgeons to Maternity Hospital, Edinburg, say:

On the whole, the results of anesthetic midwifery, as observed by us in the hospital, have been perfectly satisfactory; and we can confidently state that the recoveries have been altogether more perfect and speedy than before. This has been remarked in so great a proportion of the cases, that there can be no doubt whatever of the truth of the observation. Besides the increased rapidity of recovery, we have noticed the almost entire absence of those uncomfortable feelings of fatigue, languor, and shivering, and of that shattered feeling which so frequently comes upon the mother immediately after an ordinary delivery. Instead of this, we have found the mother almost invariably awake from the anesthetic sleep comparatively fresh, easy, and cheerful. Not unfrequently the anesthetic has been found to change, without an intermission, into a natural sleep, which may continue for an hour or two.

Further, there have been, since the introduction of chloroform into the practice of the hospital, far fewer than formerly of those violent attacks of rigors, ephemeral fevers or weeds, and abdominal pains, which are so common in most crowded hospitals, forming a class of cases which used formerly to cause much anxiety, and was a common cause of the mother's being detained in the hospital after the usual fortnight allowed for recovery. In fact, since using chloroform, there have been scarcely any women detained in the house by these causes, and much less Dover's powder, calomel and opium, abdominal fomentations, &c., have been used.

The women have been, invariably, found deeply grateful for the relief to their sufferings afforded by the anesthetic influence of chloroform.

Dr. Keith says:

I have employed chloroform in every case of labor under my care, since its introduction, with one exception; and also in almost every case to which I have been called in by other practitioners. * * * I can state most positively that I have seen no serious symptoms, which could be traced to the chloroform, in any one case, either as affecting the mother or the child. Most of the mothers have made uncommonly good recoveries. Those who have had children previously, have, almost without exception, stated to me, that they felt very decidedly stronger after delivery than on former occasions. In two cases the recovery was rather slow, but this was owing to the patients having been in a very delicate state during pregnancy—and, in both instances, I considered the chloroform was of very great service, by saving their strength. In *none* of these cases was the child still-born.

Says Dr. Moir of Edinburg :

Since the beginning of December, I have, with a very few exceptions, used chloroform in the course of my midwifery practice, and I have not met with a single case where any unpleasant effects, either to the mother or child, can be traced to its use.

Hear Dr. Malcolm :

Since November last I have employed chloroform in above thirty cases of labor, and with the most satisfactory and delightful results. A majority of these were first labors. I have kept my patients under it for periods varying from half an hour to six hours, and have never found the slightest unpleasant effects result from its use. All the children have been born alive, and are at this moment in perfect health, with the exception of one that died when about a month old, of a sudden and severe attack of dysentery. All the mothers have made recoveries with rapidity and completeness, far above the average which I had previously observed in my practice. This has struck me as the more remarkable, seeing a large proportion of my patients were primiparous ; and I can only attribute this result to the entire absence of suffering and shock to the nervous system which is effected by the use of chloroform.

Dr. Burns of Edinburg says:

I regret that I cannot give you the number of cases of labor in which I have exhibited the chloroform, but I may state that I have given it repeatedly, and have not seen any bad consequences either to the mother or child result from its use.

All the mothers made rapid recoveries, and the children did not appear to suffer from its use.

I have given the chloroform in three or four cases of adherent placenta where the uterus was firmly contracted —and had far less difficulty in extracting it than I have experienced in similar cases where the chloroform was not exhibited.

Dr. Purdie speaks in this fashion:

I have now used chloroform in seventeen cases, which I have noted, and in every instance with decided effect, not merely by lessening suffering, but I am perfectly convinced, by the most careful observation, by shortening the duration of labor. The pains have never in my experience been interfered with, except by rendering them quicker, and far more effectual.

And then Dr. Cumming:

I am quite satisfied that, if properly given, it acts as a calmative; and I believe, from what has passed under my observation, that very many of what are called exceptional cases are not so in reality, but appear to be such from error in the mode of administration, and that further experience will amply demonstrate the truth of this.

In short, I am, unfortunately for the appearance of veracity, compelled to say, that all my cases hitherto have been so successful, the recoveries so uniformly good, and the satisfaction on the part of the patient (I may add also my own) so great, that I am rapidly approaching to, if indeed I have not already arrived at, the conviction, that, *if there be any sin connected with chloroform, it is chargeable on those who refuse to administer it.*

I may add, that not one of those patients who have already inhaled it will ever be denied it in any subsequent pregnancy, as they have repeatedly assured me; and certainly I shall not attempt to keep it from them, and that not more for their sake than my own.

Dr. Grigor of Nairn uses this strong language:

Dr. Allan of Forres and myself would as soon think of going to an obstetric case without our chloroform phial, as we would of going to bleed a patient without a lancet. In this quarter, doctors are only called in when things are going wrong, or in extreme cases; so that, since your grand discovery, he and I have only used it in about twenty-four cases, in all which it came up to all you have written about it,—no still-born children—mothers recovering well—fewer after-pains, &c. &c. One of my cases was a first child, the mother nearly forty-eight years of age, weakly in constitution, and of small formation. Had it not been for the chloroform, I do think she would have sunk.

Dr. Protheroe Smith says:

I have records in my own practice and that of my friends, of upwards of 125 cases of anesthetic labor; and with one exception, all have done well. In several thus treated, no hemorrhage has ensued, though in previous labors there was flooding. In nearly all, the getting up has been more speedy, requiring no aid of opiates and purgatives; and it is my sincere conviction that chloroform lessens the chance of puerperal inflammation and fever.

And so say hundreds and thousands of others—men equally capable of observing and candid in reporting—upon whose judgment, accuracy and sincerity, implicit reliance may be placed. In the New World, as well as the Old, witnesses—not so numerous as yet, but equally clear, decisive, and united in their testimony—have arisen. Some of these, like Dr. Simpson, use it in every case,—others, mainly in the more difficult and protracted labors, or where manual or instrumental assistance is required. Dr. Miller, the much and justly esteemed Prof. of Obstetrics in our city University, has repeatedly used it in these latter circumstances, and always with the happiest results. Nay, so overwhelming now is the testimony in favor of the superinduction of anesthesia in many, if not in all cases of labor, that the "Committee on Obstetrics," in their late Report to the National Convention, actually propound the question, in all seriousness, "*Can anesthetics be rightfully withheld in midwifery?*

The opponents of the practice of etherization say, yes —and why?

The grounds of objection are numerous—medical, moral, social and *religious.* Our limits will not allow us to answer these at length, or even to glance at them. We must refer our readers to this collection of Dr. Simpson's essays on this interesting topic, for a complete refutation of everything which has thus far been urged against this practice. We bespeak for this volume a careful perusal, and for the whole subject of anesthesia in all its branches and in all its applications, a fair, candid and patient *trial;* —and such trial, we are sure, will not only end in its most honorable acquittal of all the charges that have been so hastily and groundlessly preferred against it, but will place in brighter light and clearer view its incalculable value.

We should be very careful how, on purely theoretical grounds, or hasty prejudices, or insufficient tests, we reject an agent which may be made subservient to the high purposes of humanity. A substantial fact is worth a thousand flimsy, finespun theories; and prejudices must yield finally to the light of reason, and, above all, to the teachings of experience. 'Tis now but a half century since the illustrious Jenner first made a public announcement of his plan of stopping the ravages of the small pox. Public attention was called to the fact that from thirty to fifty thousand people died annually of that loathsome disease in England alone. A simple, safe and efficacious means of arresting its progress and stripping it of its terrors was offered to the world. In what spirit was it met? Societies and associations were formed to put down the "unholy practice of vaccination." The vaccination of the children of the poor was pronounced "a gross violation of religion, morality, law and humanity." It was accused of "poisoning the pure blood of British freemen, and entailing on them and their posterity, beastly diseases." Nay, of those who submitted to it, the women were said to "cough like cows," and the men to "bellow like bulls." The king was called on to prohibit the horrible practice; the parliament was petitioned to suppress it by actual legislation; the people were entreated to rise up in arms against the wretches who were engaged in this most atrocious villiany; the pulpit rang with denunciations of "impiety," "madness," "profanity;" and sabbath after sabbath, from every church and hillside in the land, resounded the most terrific anathemas pronounced on these "miscreants." But calmly and steadily they pursued the even tenor of their way, until now nine-tenths of all the civilized people on the globe are vaccinated. The mother now needs no urging from the physician—she imperiously demands protection for her infant. At the lowest calculation, *five hundred thousand fewer people* perish annually from small pox than there did fifty years ago—and it is not too much to hope that ere another century shall have rolled away this loathsome disease will be altogether exterminated.

Until about three hundred years ago, after amputations, (which were far more frequent then than now), or in cases of accidents, or under any circumstances where it was necessary to control a hemorrhage, surgeons universally had recourse to a red hot iron, or the potential cautery, or boiling pitch, which were applied to the raw and bleeding wounds—producing tortures inconceivable and deaths innumerable. "The horrors of the patient, and his ungovernable cries," says the late Mr. John Bell, (*Principles of Surgery, vol.* 1, *p.* 212,) "the hurry of the operators and assistants, the sparkling of the heated irons, and the hissing of the blood against them, must have been terrible scenes; and surgery must, in those days, have been a horrible trade."

Ambrose Paré proposed a simple, safe, and, comparatively speaking, painless method of avoiding this shocking and dangerous practice, viz: the application of ligatures to the arteries. And how was *his* proposition met? "The College of Physicians of Paris attacked Paré for his proposed new practice. They attempted, by the authority of the French Parliament, to suppress the publication and dissemination of his observations; and, for nearly a long century afterwards, some of the hospital surgeons continued, with the characteristic obstinacy of the profession, to prefer cauterizing bleeding arteries 'with *all* the ancients,' rather than simply tie them 'after the manner of a few ignorant and presumptuous moderns!'"

These and other facts of like import, so abundant in the history of medical science, are fraught with warning and yet with encouragement. They warn us that new doctrines and new remedies are not to be despised and rejected because they are new; and at the same time they encourage us to hope that whenever a valuable, although novel, weapon is devised to combat our old enemy, all the opposition and reproach it may meet with, will be finally overthrown and trampled under foot. For who now does not resort to vaccination? Who would not hiss and deride the surgeon who would prefer boiling pitch to the ligature as a means of arresting hemorrhage? Whose names can the medical man point more proudly to, than those of Edward Jenner and Ambrose Paré?

